# Naturally Acquired Human *Plasmodium knowlesi* Infection, Singapore

**DOI:** 10.3201/eid1405.070863

**Published:** 2008-05

**Authors:** Oon Tek Ng, Eng Eong Ooi, Cheng Chuan Lee, Piao Jarrod Lee, Lee Ching Ng, Sze Wong Pei, Tian Ming Tu, Jin Phang Loh, Yee Sin Leo

**Affiliations:** * Tan Tock Seng Hospital, Singapore; † DSO National Laboratories, Singapore; ‡ Singapore Armed Forces Headquarters Medical Corps, Singapore; § Environmental Health Institute, Singapore

**Keywords:** Malaria, zoonoses, parasites, dispatch

## Abstract

We report a case of naturally acquired *Plasmodium knowlesi* in Singapore, a malaria-free country. Diagnosis was confirmed by PCR with validated species-specific primers. In industrialized countries, free-ranging primates are a potential source of *P. knowlesi* human infection. *P. knowlesi* infection is a differential diagnosis of febrile illness acquired in Singapore.

## Abstract 2

Naturally Acquired Human *Plasmodium knowlesi* Infection, Singapore

*Plasmodium knowlesi* is one of the simian malarias that causes human infection (*1**,**2*). All 6 published reports of naturally acquired *P. knowlesi* infection were in rural settings with the largest case series being reported from East Malaysia (*3**–**8*). *P. knowlesi* is commonly misidentified as *P. malariae* since the blood stages are morphologically similar on microscopy, and molecular methods of detection are necessary for accurate diagnosis (*5**,**8*).

Singapore is an urban city-state, which was declared free of human malaria by the World Health Organization in 1982 (*9*). However, we report a case of locally acquired *P. knowlesi* malaria, which indicates that this emerging zoonotic parasite should be considered as an etiologic agent of acute febrile illness acquired in Singapore, the southern-most locale reported thus far.

## The Case

A previously healthy 20-year-old soldier in the Singapore Army sought treatment on April 28, 2007. He had had a fever for 4 days, along with myalgia, anorexia, nausea, and occasional vomiting. For a year leading up to his illness, he had trained in a forested area inhabited by the long-tailed macaque (*Macaca fascicularis*) in Lim Chu Kang, northwestern Singapore. His only travel out of Singapore was a 3-week training visit to a non–malaria-endemic foreign country in September 2006 and another visit to Bukit Batok Nature Reserve in western Singapore, an area with monkeys (*M. fascicularis*) 1 month before onset of symptoms. On initial examination, his temperature was 39.5°C with a pulse rate of 106 beats/min. He was lethargic with tender hepatomegaly. Laboratory investigations showed thrombocytopenia (platelet count 66 × 10^9^/L), hyperbilirubinemia (bilirubin 33 mmol/L [reference level 7–31 mmol/L]), and mild transaminitis (alanine transaminase 64 U/L [reference level 17–63 mmol/L] and aspartate transaminase 67 U/L [reference level 15–41 mmol/L]).

**Figure 1:**
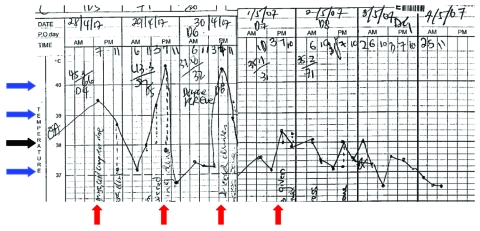
Patient’s temperature chart showing fever spikes 24 h apart at approximately 7 PM daily (red arrow). The black arrow denotes 38°C, and each blue arrow denotes a difference of 1°C from the neighboring arrow.

Initial diagnosis was dengue fever, which is endemic in Singapore. The patient experienced daily fever spikes from 39.5°C to 40.4°C (Figure 1). When fever persisted (40.4°C on day 6 of his illness, hospital day 3), the clinical picture was atypical for dengue fever. Blood films for malaria parasites were ordered, because introduced cases of malaria have been reported in Singapore (*10*). Microscopy showed *Plasmodium* parasitemia of 0.2% (equivalent to 7,700 parasites/mmol/L blood) with morphologic features consistent with *P. malariae*. Results of dengue reverse transcription–PCR (RT-PCR) on serum, 2 sets of blood cultures, and *Rickettsia* *typhi* serologic testing were negative. Results of a chest radiograph and ultrasound of the abdomen were normal.

Oral chloroquine was started with an initial dose of 600 mg base, followed by 300 mg base 6 h later and another 2 doses over the next 2 days. He defervesced rapidly; blood smears were negative 3 days after chloroquine therapy. At 2 weeks follow-up, he was clinically well.

Because *P. malariae* infection was not consistent with the clinical findings of the initial examination, we investigated further to determine the etiology of this case. End-point nested *Plasmodium* genus- and species-specific nested PCR carried out on DNA extracted from whole blood samples were positive for *Plasmodium* sp. but negative for the 4 species that cause human malaria (Table) (*11*). Similarly, the sample was negative on real-time PCR for the 4 human parasites (*12*). *P. knowlesi* species-specific PCR resulted in a 153-bp fragment indicative of *P. knowlesi* (*5*). This 153-bp PCR product was directly sequenced and verified in the BLAST database (www.ncbi.nlm.nih.gov/blast/Blast.cgi) to match only *P. knowlesi* small subunit ribosomal RNA (SSU rRNA).

**Table:** Primers used for the PCR investigation of the clinical sample from Singapore*

Primers	Forward	Sequence (5′ → 3’)	Reverse	Sequence (5′ → 3’)	Results
Nest 1, genus specific primers					
Genus specific (*11*)	rPLU 1	TCA AAG AAT AAG CCA TGC AAG TGA	rPLU 2	TAC CCT GTT GTT GCC TTA AAC TCC	+
Nest 2, genus- and species-specific primers					
Genus specific (*11*)	rPLU 3	TTT TTA TAA GGA TAA CTA CGG AAA AGC TGT	rPLU 4	TAC CCG TCA TAG CCA TGT TAG GCC AAT ACC	+
Plasmodium knowlesi specific (*5*)	Pmk8	GTT AGC GAG AGC CAC AAA AAA GCG AAT	Pmkr9	ACT CAA AGT AAC AAA ATC TTC CGT A	+
*P. vivax* specific (*11*)	rVIV1	CGC TTC TAG CTT AAT CCA CAT AAC TGA TAC	rVIV2	ACT TCC AAG CCG AAG CAA AGA AAG TCC TTA	–
P. falciparum specific (*11*)	rFAL1	TTA AAC TGG TTT GGG AAA ACC AAA TAT ATT	rFAL2	ACA CAA TGA ACT CAA TCA TGA CTA CCC GTC	–
P. malariae specific (*11*)	rMAL1	ATA ACA TAG TTG TAC GTT AAG AAT AAC CGC	rMAL2	AAA ATT CCC ATG CAT AAA AAA TTA TAC AAA	–
*P. ovale* specific (*11*)	rOVA1	ATC TCT TTT GCT ATT TTT TAG TAT TGG AGA	rOVA2	GGA AAA GGA CAC ATT AAT TGT ATC CTA GTG	–

*PCR was carried out at the Environmental Health Institute and cycling conditions used were as described in the references shown in parentheses.  +, positive; –,negative.

**Figure 2:**
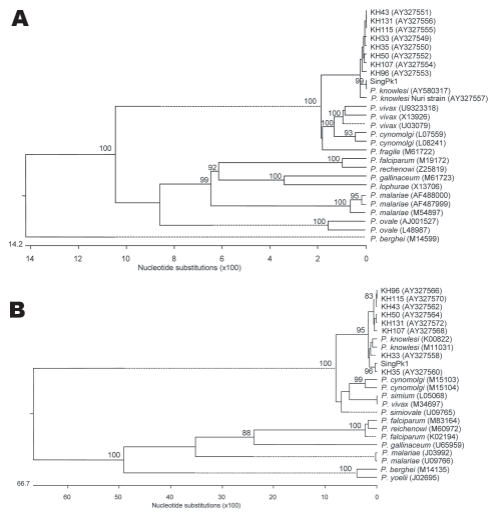
Phylogenetic trees comparing our case sample (denoted as SingPk1) with other *Plasmodium* species, based on SSU rRNA (A) and *csp* (B) sequences. Species and sequences used were selected to match those previously reported (*5*). Figures on the branches are bootstrap percentages based on 1,000 replicates, and only those above 80% are shown. GenBank accession numbers are in parentheses.

We confirmed the pathogen by using previously described approaches to compare the sequences of the 5′ and 3′ ends of the circumsporozoite protein (csp) gene (*13*), as well as the gene encoding of the sSSU rRNA (*5*) in our case sample, to other *Plasmodium* parasites. Sequences were obtained by direct sequencing of PCR products and aligned by using the ClustalW method (EMBL-EBI, Hixton, Cambridge, UK); we constructed phylogenetic trees by using the MegAlign software (DNASTAR Inc, Madison, WI, USA). The case sample (denoted as SingPk1) clustered with other *P. knowlesi* isolates and is clearly distinct from other *Plasmodium* species (Figure 2).

## Conclusions

We describe an unequivocal case of *P. knowlesi* infection supported by clinical findings and laboratory diagnostics classic for this pathogen. Similar to our patient, the classic scenario that raises the suspicion of *P. knowlesi* infection is a blood smear consistent with *P. malariae* but with parasitemia exceeding 5,000 per mmol/L blood, daily fever spikes, and pronounced symptoms, features atypical for *P. malariae* infection (*1**,**5*). The daily fever spike is due to the *P. knowlesi* 24-hour asexual life cycle, the shortest of all primate malarias (*8*). *P. malariae* has a 72-hour asexual life cycle and manifests as chronic, asymptomatic infection with low level parasitemia (*5*).

As in this case, *P. knowlesi* is commonly mistaken for *P. malariae* by microscopy due to similarity of the blood stages (*5*). *P. knowlesi* can be misidentified as *P. falciparum* if only ring forms are identified (*5*). The *P. knowlesi*–specific primers used by both independent laboratories have previously been shown not to detect any of the 4 *Plasmodium* species that cause human infection or the 3 agents that cause simian malaria: *P. cynomolgi*, *P. fieldi,* and *P. fragile* (*5*). PCR detection using *P. knowlesi*–specific primers, followed by sequencing and phylogenetic analyses of the csp and SSU rRNA genes confirmed *P. knowlesi* infection in our patient. This report extends the range of natural *P. knowlesi* human infection from East Malaysia, peninsular Malaysia, Thailand, and Myanmar to Singapore, an industrialized country that had been declared malaria-free by WHO (*3**–**8*).

Our patient likely acquired the infection in the forested area in Lim Chu Kang where he had been training for the entire year before his illness. Experimental *P. knowlesi* studies show a prepatent period of 9–12 days in humans (*14*). *P. knowlesi* has no liver hypnozoite stage and does not cause relapse (*1*). The patient’s pevious overseas travel 7 months before and his visit to Bukit Batok Nature Reserve a month before onset of illness are beyond the incubation period.

*P. knowlesi*’s natural hosts are the macaques, *M. fascicularis* and *Macaca nemestrina* (*1*). Notably, the first studies on *P. knowlesi* were on a parasite isolated from a macaque imported into India from Singapore (*2*). *M. fascicularis* and *Presbytis femoralis* are the 2 native monkeys in Singapore, with *M. fascicularis* being the only species in Lim Chu Kang and Bukit Batok Nature Reserve (*15*). Mosquitoes of the *Anopheles leucophyrus* group have been identified as vectors of *P. knowlesi* and are present in surrounding countries in southeast Asia (*1**,**8*). Studies are ongoing to determine potential mosquito vectors and whether macaques are hosts of *P. knowlesi* in Singapore.

Our patient’s condition was diagnosed within 6 days of illness, and the infection responded rapidly to oral chloroquine. Although most patients’ infections respond well to antimalarial agents, 4 fatal cases of *P. knowlesi* infection were reported recently in patients ages 39 to 69 years, whose conditions were all diagnosed within 7 days of symptom onset (*8*). Common clinical features included fever, abdominal pain, thrombocytopenia (platelet count <30 × 10^9^/ μL), renal impairment, and jaundice. All of the patients received a misdiagnosis of *P. malariae* infection.

*P. knowlesi* infection should be considered as an etiologic agent of malaria acquired in Singapore, particularly in cases with daily fever spikes and blood smears suggestive of *P. malariae*. Epidemiologic studies into the parasite’s reservoir and mosquito vector will be important in the prevention of this emerging zoonotic disease.
